# An updated meta-analysis of the prognostic value of decreased E-cadherin expression in ovarian cancer

**DOI:** 10.18632/oncotarget.20885

**Published:** 2017-09-14

**Authors:** LiLi Yu, Xiaoli Hua, Yu Yang, Ke Li, Qilin Zhang, Lixiu Yu

**Affiliations:** ^1^ Department of Obstetrics and Gynecology, Union Hospital, Tongji Medical College, Huazhong University of Science and Technology, Wuhan 430022, PR China; ^2^ Department of Pharmacy, Union Hospital, Tongji Medical College, Huazhong University of Science and Technology, Wuhan 430022, PR China

**Keywords:** ovarian cancer, E-cadherin, prognosis, updated meta-analysis

## Abstract

Decreased epithelial cadherin (E-cadherin) expression is hypothesized to be related to poor prognosis of ovarian cancer, but the predictive value is still inconsistent. We conducted an updated meta-analysis with a total of 16 studies enrolling 1720 patients to estimate the prognostic value of decreased E-cadherin expression in ovarian cancer. Reduced expression of E-cadherin was significantly associated to poor overall survival (HR = 1.74, 95% CI: 1.40–2.17) and progression-free survival (HR = 1.45, 95% CI: 1.12–1.86) with a large heterogeneity for overall survival. In addition, we found that decreased expression of E-cadherin was significantly correlated with International Federation of Gynecology and Obstetrics grade (HR = 3.74, 95% CI: 2.24–6.23), E-cadherin membranous (HR = 1.47, 95% CI: 1.01–2.14), pathologic grade (HR = 1.41, 95% CI: 1.01–1.97), residual tumor size (HR = 2.72, 95% CI: 1.99–3.72), and surgery (HR = 3.21, 95% CI: 1.19–8.67). Our finding suggests that decreased E-cadherin expression may be a predictor of poor ovarian cancer prognosis.

## INTRODUCTION

Ovarian cancer threatens women's health worldwide as a lethal disease that is challenging to diagnose in early stages [1, 2]. Although progress has been made in diagnostics and treatments of ovarian cancer, the prognosis of ovarian cancer patients is far from optimistic [2, 3]. The International Federation of Gynecology and Obstetrics (FIGO) stage, histotype and grade of differentiation are recognized as classical prognostic factors, but they cannot accurately predict the prognosis of ovarian cancer [4, 5]. However, studies have proved that many biomarkers are involved in the progression of ovarian cancer [6], identification and validation of prognostic factors can complement well-established clinical histopathology analysis with the aim of improving future treatments [7].

E-cadherin is a calcium dependent transmembrane glycoprotein, located in chromosome 16q22.1. Inhibition of E-cadherin function can lead to reduced cell proliferation and viability [8]. Decreased expression of E-cadherin can destroy the intracellular junction so that epithelial cells acquire the ability to migrate [9]. Therefore, impaired function of E-cadherin could lead to invasive potential and migration of malignant epithelial tumors. Previous studies have suggested that decreased expression of E-cadherin is closely related to the occurrence, differentiation, invasion, metastasis, and prognosis of tumors in ovarian, breast, gastric, and prostate cancer [9–13]. The possible mechanisms may be E-cadherin gene mutation, E-cadherin promoter hypermethylation, suppression of RNA transcription, or matriptase activation [14, 15]. Studies also suggested that decreased E-cadherin expression was associated with high histological grade and deep myometrial invasion [16, 17]. However, some other studies drew different conclusions [18–20], so associations between decreased expression of E-cadherin and ovarian cancer prognosis are still debated. Previous reviews, including meta-analyses, did not explore associations between decreased expression of E-cadherin and progression-free survival (PFS) and histological grade in depth [21, 22]. Considerable progress has been made in this area [23–26], so we conducted an updated meta-analysis to evaluate the relationship between E-cadherin and ovarian cancer prognosis comprehensively.

## RESULTS

### Search results

**Figure 1:**
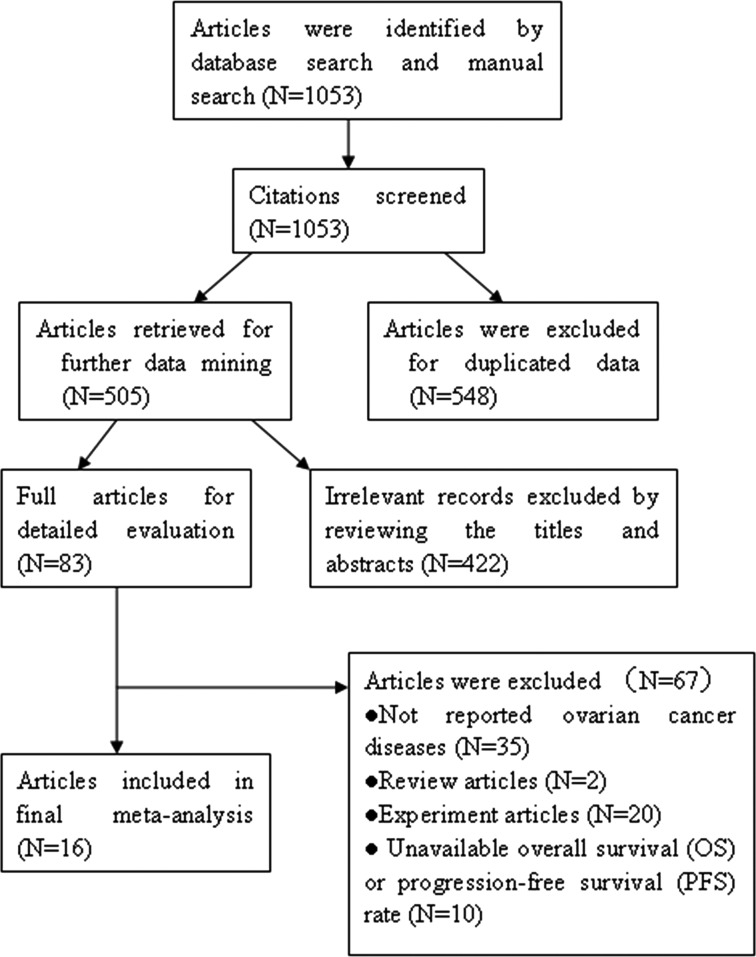
The flow diagram of retrieval in this study

As shown in Figure 1, a total of 1053 citations were identified. Of these, 548 articles were removed for duplicated data. Through reviewing titles and abstracts, 422 articles were excluded due to irrelevant publications on E-cadherin expression in ovarian cancer. After systematically reading the remaining studies, 67 articles were excluded because the studies were *in vitro* or non-human studies, reviews, or did not report overall survival (OS) or progression-free survival (PFS) in ovarian cancer. A total of 16 articles with 1720 ovarian cancer patients [18–20, 23–35] were determined eligible.

### Study characteristics

The main characteristics of the eligible studies are documented in Table 1. Of the 16 articles published from 1997 to 2017, thirteen articles were high-quality studies (NOS scores ≥ 7.0). Seven studies presented the OS and PFS, while nine studies present OS only. The level of E-cadherin expression in ovarian cancer tissues was frequently detected by immunohistochemistry (IHC). The hazard ratio (HR) value was obtained directly or from survival curves of the studies.

**Table 1:** Study characteristics

First author	Country	Published year	Mean age (year)	FIGO stage	Cases	Mean Follow-up (year)	Cut-off level (%)	Method	Survival type	HR estimation	NOS
Sundov D. [23]	Croatia	2017	55	I-VI	81	NA	10	IHC	OS	Directly obtained	7
Juan W. [24]	China	2016	53	I-VI	257	3	NA	IHC	OS /PFS	Directly obtained	7
Li X. [25]	China	2016	47	I-VI	43	2.12	20	IHC	OS/PFS	Directly obtained	7
Liew PL. [26]	Taiwan	2015	54.3	I-VI	108	4.13	10	IHC	OS/DFS	Directly obtained	7
Bodnar L. [27]	Poland	2014	54	I-VI	61	NA	10	IHC	OS/ PFS	Directly obtained	8
Taskin S. [28]	Ankara	2012	58.63	I-VI	30	2.81	25	IHC	OS	Directly obtained	6
Huang KJ. [18]	China	2012	NA	NA	136	2.13	5	IHC	OS	Directly obtained	7
Dian D. [19]	Germany	2011	60.35	I-VI	100	13	25	IHC	OS/ PFS	From curves	7
Ho CM. [29]	China	2010	51	II-VI	58	2.72	10	IHC	OS/PFS	Directly obtained	6
Shim HS. [30]	Kroea	2009	50.2	II-VI	72	NA	25	IHC	OS	From curves	7
Blechschmidt K. [31]	Germany	2008	63	III, IV	48	4.58	10	IHC	OS	Directly obtained	8
Scholten AN. [32]	Netherlands	2006	NA	I-VI	225	11.6	25	IHC	OS	Directly obtained	7
Cho EY. [33]	Korea	2006	NA	I-VI	95	2.67	10	IHC	OS	From curves	6
Voutilainen KA. [20]	Finland	2006	62	I-VI	282	12	5	IHC	OS /PFS	From curves	8
Faleiro-Rodrigues C. [34]	Portugal	2004	56	I-VI	104	6	10	IHC	OS	Directly obtained	8
Darai E. [35]	France	1997	53.5	I-III	20	24	10	IHC	OS	From curves	7

Note: NA: Not available; HR: Hazard ratio; FIGO: International Federation of Gynecology and Obestetrics; IHC: Immunohistochemistry; OS: Overall survival; PFS: Progression-free survival; NOS:Newcastle-Ottawa quality assessment scale.

### Influence of reduced E-cadherin expression on OS and PFS

**Figure 2:**
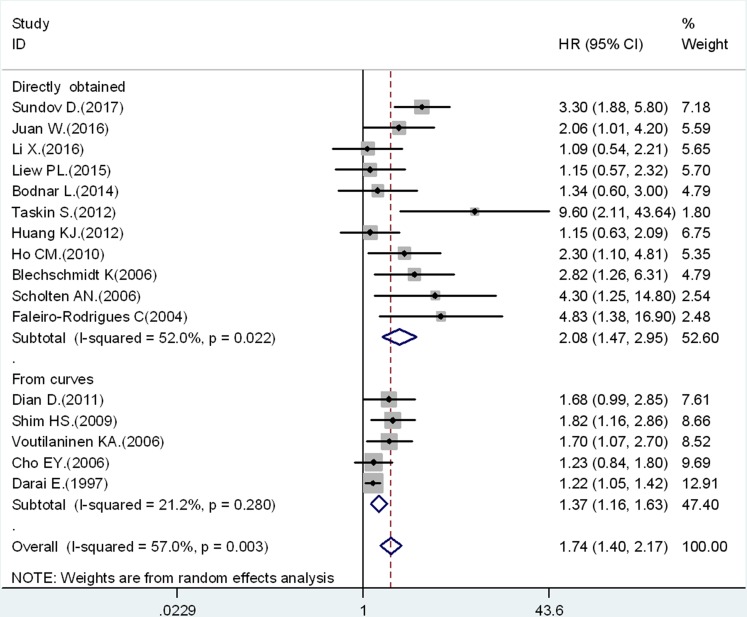
Association between decreased E-cadherin expression and OS stratified by HR estimation: directly obtained and from curves

Pooled results of the 16 studies suggested that reduced expression of E-cadherin was significantly associated with OS (HR = 1.74, 95% CI: 1.40–2.17) (Figure 2). Thus, ovarian cancer with reduced E-cadherin expression had a higher risk of mortality, but significant heterogeneity (*I*^2^ = 57.0%, *p* = 0.003) was observed across the studies, then random effect model was used. We performed further subgroup analysis and meta-regression to explain significant heterogeneity from six distinct sources, which are listed in Table 2. HR extracted directly from the articles was 2.08 (95% CI: 1.47–2.95) with significant heterogeneity (*I*^2^ = 52.0%, *p* = 0.022) (Figure 2; Directly obtained). The pooled HR obtained from the Kaplan–Meier curves was 1.37 (95% CI: 1.16–1.63) with no significant heterogeneity (*I*^2^ = 21.2%, *p* = 0.28) (Figure 2; From curves). We calculated pooled HRs for OS and PFS from multivariate or univariate criteria separately. In the multivariate HR estimation group, the pooled HR for OS was 2.30 (95% CI: 1.62–3.26, *I*^2^ = 71.6%, *p* < 0.01), and the pooled HR for PFS was 2.52 (95% CI: 1.33–4.78, *I*^2^ = 0%, *p* = 0.33). Similarly, in the univariate HR estimation group the pooled HR for OS was 1.38 (95% CI: 1.11–1.70, *I*^2^ = 1.4%, *p* = 0.42), and the pooled HR for PFS was 1.29 (95% CI: 0.99–1.69, *I*^2^ = 0%, *p* = 0.50). The results indicated that there were significant associations between reduced expression of E-cadherin and OS and PFS, except for the univariate HR estimation group for PFS. The results further confirmed that patients with decreased expression of E-cadherin had poor OS in ovarian cancer. Considering the evaluation criteria of IHC, the pooled HR was 1.72 (95% CI: 1.32–2.24) in the percentage group, 2.50 (95% CI: 1.39–4.51) in the semi-quantitative group, and 1.25 (95% CI: 1.08–1.43) in the combined group. These results also suggested that the correlation between reduced expression of E-cadherin and poor OS was significant. Meta-regression analysis showed that there was no statistically significant difference among subgroups (*p* = 0. 364). In other stratified analyses by year, nation, article quality, and histological type, we found no statistically significant difference among subgroups as presented in Table 2.

**Table 2:** The pooled data on overall survival of meta-analysis

Subgroup analysis	Pooled HR (95% CI)		Meta-regression (*p* value)	Heterogeneity	
	Random-effect model	Fixed-effect model		I2 value (%)	*p* value
Year			0.114		
≤ 2010	1.69 (1.30–2.20)	1.37 (1.21–1.55)		59.40	0.016
> 2010	1.77 (1.25–2.51)	1.74 (1.38–2.19)		52.50	0.032
Country			0.094		
Asia	1.55 (1.24–1.94)	1.46 (1.25–1.71)		33.80	0.15
Non–Asia	2.26 (1.42–3.61)	1.43 (1.23–1.66)		72.0	0.001
HR estimation			0.036		
From curves	1.37 (1.16–1.63)	1.33 (1.17, 1.50)		52.0	0.02
Directly obtained	2.08 (1.47–2.95)	1.92 (1.53–2.40)		21.10	0.28
Scoring criteria			0.364		
Percentage	1.72 (1.32–2.24)	1.69 (1.37–2.09)		29.9	0.179
Semiquantitative	2.50 (1.39–4.51)	2.46 (1.70–3.55)		56.2	0.077
Combined	1.25 (1.08–1.43)	1.25 (1.08–1.43)		0.2	0.367
NOS			0.819		
6	2.33 (0.95–5.73)	1.54 (1.11–2.14)		75.3	0.017
7	1.62 (1.23–2.12)	1.37 (1.21–1.55)		58.3	0.014
8	2.01 (1.31–3.09)	1.93 (1.37–2.72)		24.9	0.26
Histological type			0.425		
Serious	1.84 (1.33–2.53)	1.81(1.37–2.40)		18.4	0.29

**Figure 3:**
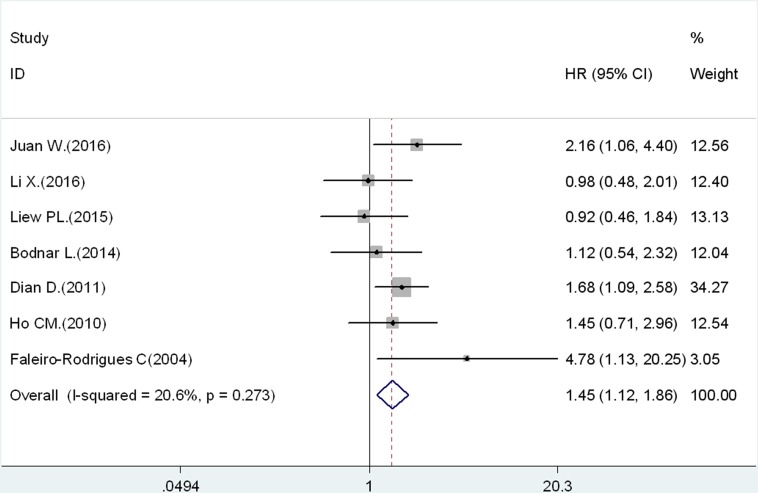
Results of association between decreased expression of E-cadherin and PFS

The PFS analysis was based on 7 studies. The pooled results indicated that decreased expression of E-cadherin could prefigure poor PFS in ovarian cancer (HR = 1.45, 95% CI: 1.12–1.86, Figure 3). There was no significant heterogeneity (*I^2^* = 20.6%, *p* = 0.273).

### Association between reduced E-cadherin expression and clinicopathological characteristics

The pooled results for reduced E-cadherin expression and clinicopathological characteristics are illustrated in Table 3. The results indicated that decreased expression of E-cadherin was significantly associated with FIGO stage (III–IV vs. I–II: HR = 3.74, 95% CI: 2.24–6.23), surgery (suboptimal vs. optimal, HR = 3.21, 95% CI: 1.19–8.67), residual tumor (≥ 1cm vs. < 1 cm, HR = 2.72, 95% CI: 1.99–3.72), and E-cadherin membranous (negative vs. positive, HR = 1.47, 95% CI: 1.01–2.14). However, the association between decreased E-cadherin expression and lymphatic metastasis (negative vs. positive: HR = 1.40, 95% CI: 0.63–3.10) or chemotherapy (non-paclitaxel vs. paclitaxel: HR = 1.31, 95% CI: 0.31–5.57) was not significant.

**Table 3:** Associations between decreased expression of E-cadherin and clinicopathological features

Clinicopathological features	Pooled HR	95% CI for HR		*p* value	*I*^2^ value (%)	Model
		Lower	Upper			
Age (≥ 50 vs. < 50)	1.07	0.85	1.36	0.145	41.5	Random
FIGO stage (III, IV vs. I, II)	3.74	2.24	6.23	0.37	0	Fixed
E-cadherin membranous staining (negative vs. positive)	1.47	1.01	2.14	0.78	0	Fixed
Pathologic grade (G3 vs. G1, G2)	1.41	1.01	1.97	0.13	47	Random
Residual tumor (<1cm vs. > 1cm)	2.72	1.99	3.72	0.51	0	Fixed
Surgery (suboptimal vs. optimal)	3.21	1.19	8.67	0.074	68.7	Random
Chemotherapy (non-paclitaxel vs. paclitaxel)	1.31	0.31	5.57	<0.01	90.4	Random
Lymphatic metastasis (negative vs. positive)	1.40	0.63	3.10	0.01	86.6	Random

### Sensitivity analysis

**Figure 4:**
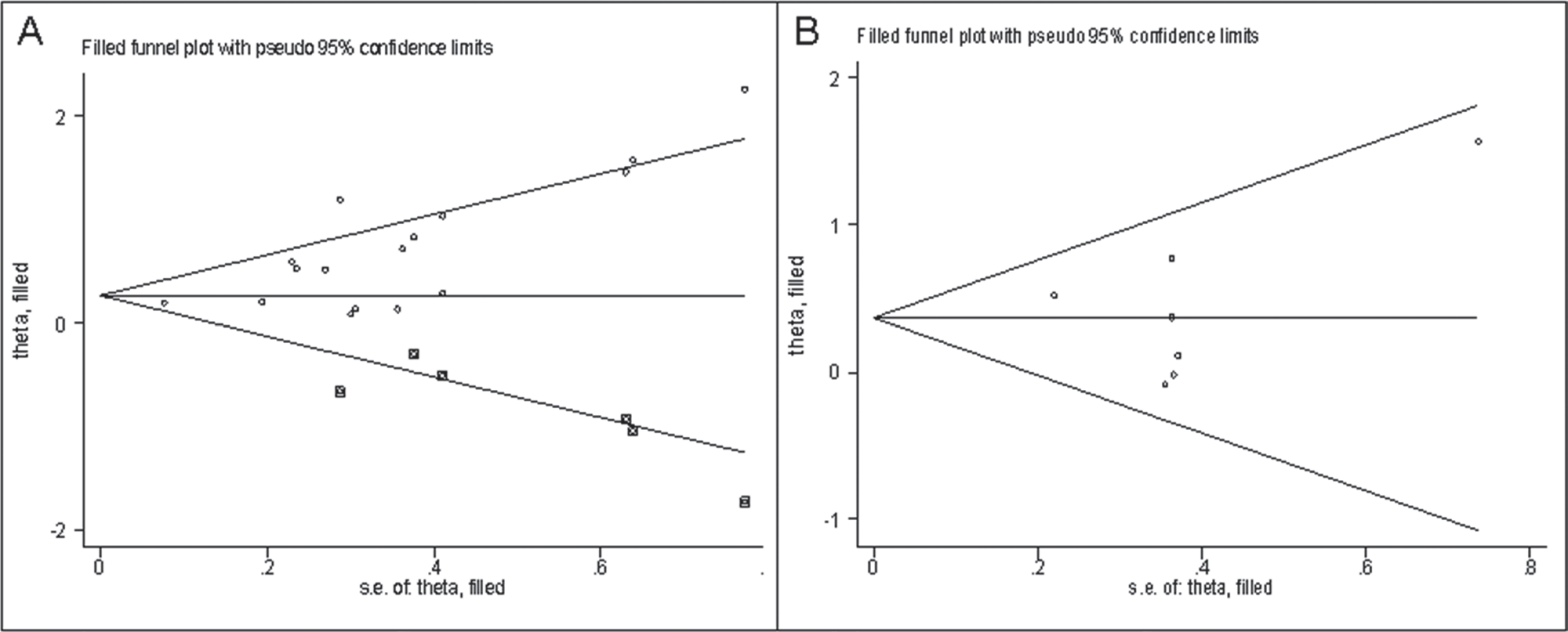
Sensitivity analysis for OS (**A**) and PFS (**B**).

Sensitivity analysis was conducted by sequential omission of each article to evaluate stability concerning OS and PFS. Exclusion of any single study did not alter the pooled HR estimation, which indicated that our results were robust (Figure 4).

### Publication bias

**Figure 5:**
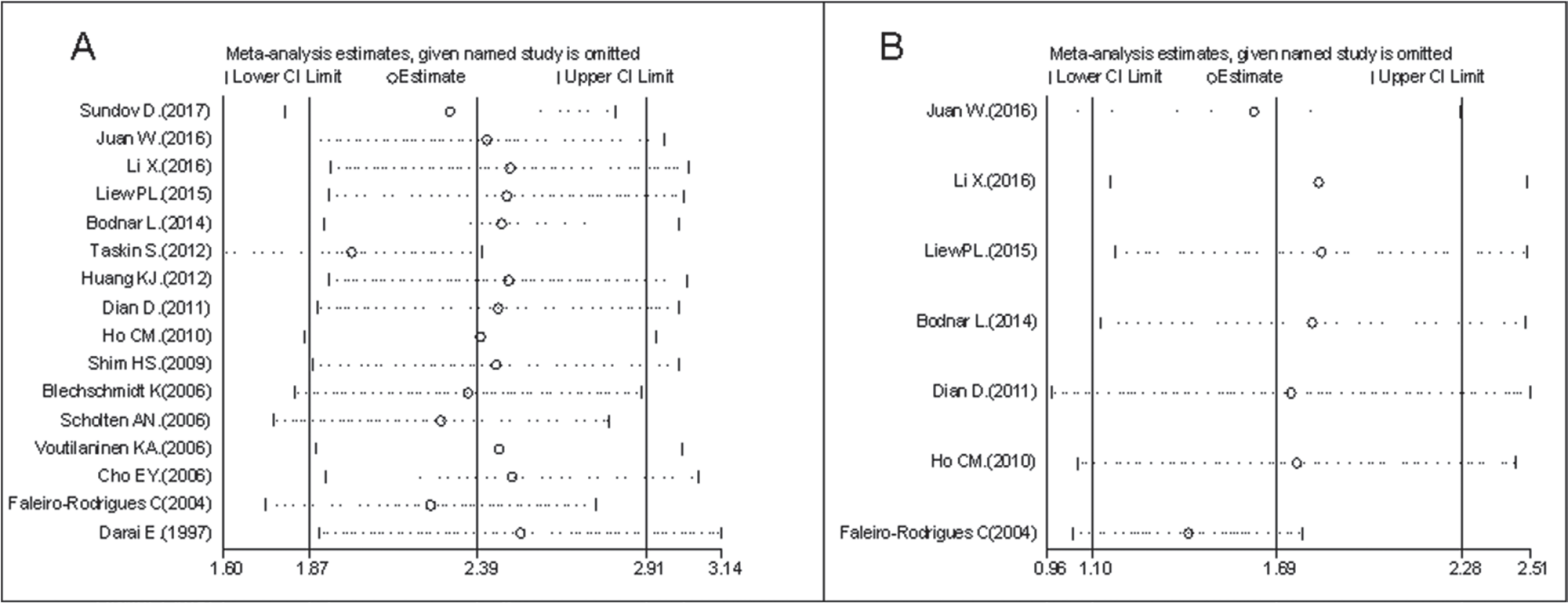
Funnel plot of publication bias for OS (**A**) and PFS (**B**).

Both Funnel plot and Egger's tests were used to evaluate the publication bias of included articles. Our results suggested that there was potential asymmetry in the studies on the association between reduced expression of E-cadherin and OS (*p* Egger = 0.002), so a trim-and-fill analysis was conducted. As shown in Figure 5A, after incorporation of six additional studies, the funnel plots were symmetrical, and E-cadherin low-expression was related to poor OS (corrected HR = 1.37, 95% CI: 1.08–1.74). Figure 5B shows that all articles lay inside the 95% CIs, with an even distribution around the vertical. Egger's tests also suggested no significant publication bias (*p* Egger = 0.70).

## DISCUSSION

We conducted a comprehensive meta-analysis toward investigating the prognostic value of decreased E-cadherin expression in ovarian cancer. A total of 16 articles [18–20, 23–35] with 1720 enrolled patients were included in this meta-analysis. The results indicated that decreased expression of E-cadherin was significantly associated with poor OS (HR = 1.74, 95% CI: 1.40–2.17, *I*^2^ = 57.0%, *p* = 0.003) and poor PFS (HR = 1.45, 95% CI: 1.12–1.86, *I*^2^ = 20.6%, *p* = 0.273), suggesting that patients with decreased expression of E-cadherin have poor prognosis in ovarian cancer. Consistent with the conclusion that low expression of E-cadherin carries a worse prognosis [26, 29], E-cadherin may be an important target for the treatment of ovarian cancer.

We also assessed the associations between decreased E-cadherin expression and clinicopathological features. The results indicated that decreased expression of E-cadherin was significantly associated with high FIGO stage (III and IV) compared with low FIGO stage (I and II) (HR = 3.74, 95% CI: 2.24–6.23, *I*^2^ = 0, *p* = 0.37). Reduced E-cadherin expression has significant association with residual tumor (≥ 1cm vs. < 1cm, HR = 2.72, 95% CI: 1.99–3.72), E-cadherin membranous staining (negative vs. positive, HR = 1.47, 95% CI: 1.01–2.14), and surgery (suboptimal vs. optimal, HR=3.21, 95% CI: 1.19–8.67). A significant association between decreased expression of E-cadherin and lymphatic metastasis (negative vs. positive: HR = 1.40, 95% CI: 0.63–3.10) or chemotherapy (non-paclitaxel vs. paclitaxel: HR =1.31, 95% CI: 0.31–5.57) was not found. Given the limited number of original studies of clinicopathological features, further studies are needed to confirm the association between decreased E-cadherin expression and clinicopathological.

To date, there is no reliable clinical predictor for ovarian cancer, but the decreased expression of E-cadherin has been reported as an important event in ovarian cancer invasion and metastasis. E-cadherin belongs to the calcium-dependent adhesion molecule protein family and is mainly distributed in the epithelial tissue [36]. The intact structure of normal ovarian surface epithelium is dependent on N-cadherin, while E-cadherin is hardly expressed. Compared with well-differentiated epithelial carcinoma the expression of E-cadherin is usually decreased or absent in poorly-differentiated ovarian carcinoma. Previous studies demonstrated that loss of E-cadherin was the most important hallmark of epithelial–mesenchymal transition, which is implicated in the dissemination, migration, and invasion of cancer cells [37, 38]. The loss of E-cadherin expression also has an inseparable association with chemotherapy resistance in cancer cells [39] and can cause tumor cells to present apparent properties of cancer stem cells [40]. However, Darai *et al*. demonstrated that decreased expression of E-cadherin was uncorrelated with cancer type, pathological grade and tumor size [35]. Point mutation and partial deletion of E-cadherin gene can also cause loss of cell adhesion while tyrosine phosphorylation in the E-cadherin-beta catenin complex can inhibit the function of E-cadherin without changing its expression. Further mechanistic research is warranted, these studies support our hypothesis that decreased of expression E-cadherin is a promising prognosis factor of survival in ovarian cancer.

Significant heterogeneity among studies (*I*^2^ = 57.0%, *p* = 0.003) was found when survival data were pooled for OS. Subgroup analysis and meta-regression analysis were performed to explore the source of heterogeneity. The results suggested that methods for HR estimation might contain variables associated with this heterogeneity. Stratified analysis showed that the heterogeneity was less than 50% (*I*^2^ = 21.2%, *p* = 0.28) in HR obtained from curves group, yet there was significant heterogeneity (*I*^2^ = 52.0%, *p* = 0.022) in HR directly extracted group. Similarly, stratified analysis by multivariate/ univariate criteria for HR estimation suggested that the heterogeneity was significant (*I*^2^ = 71.6%, *p* < 0.01) in multivariate HR group. While, there was not significant heterogeneity (*I*^2^ = 1.4 %, *p* = 0.42) in univariate HR group. Importantly, meta-regression analysis showed that there was significant heterogeneity among the HR estimation subgroup (*p* = 0.036) (Table 2). Possible reasons for the significant heterogeneity are different cut-off points and scoring systems of HR estimation because the methods of determining the intensity and quantity of E-cadherin expression varied among individual studies. For example, Ho *et al*. defined negative E-cadherin immunoexpression as 10% positive tumor cells or less, and positive E-cadherin immunoexpression was defined more than 10% positive tumor cells [29]. The E-cadherin immunoexpression of the tumors was scored semiquantitatively according to the percentage of positive tumor cells in membranous staining on a 4-point scale of 0 to 4. However, Taskin *et al*. considered strong membranous and cytoplasmic staining to be a positive result for E-cadherin. Stained cells were scored with 0% defined as 0, < 1–25% as 1, 25–75% as 2, and > 75% as 3 [28]. The above differences in individual studies might cause variances in HR estimation leading to significant heterogeneity among studies.

Although association between E-cadherin expression and ovarian cancer prognosis has been reported, the contributions of this meta-analysis are as follows: (I) more articles (16 studies vs. 9 studies) and subjects were included in our meta-analysis to provide rigorous evidence compared to a previous meta-analysis conducted in 2012 [22]; (II) stratified analyses of HR estimation, study quality, score criteria, and histological type were conducted to explore heterogeneity; and (III) associations between decreased E-cadherin expression and clinicopathological characteristics were evaluated. Our study reveals that decreased E-cadherin expression has significant associations with FIGO stage, pathologic grade, and residual tumor, which presents a new direction for future research. Moreover, the level of E-cadherin expression was reflective of some clinical characteristics.

Despite the highlights mentioned above, there are still several limitations in our meta-analysis. First of all, the sources of primary antibody and concentration of antibody used in each study varied, which could influence IHC sensitivity. Secondly, the reliability of HR obtained from available data or Kaplan–Meier curves could be affected by inaccuracies in the calculation of censored data. Thirdly, there was no uniform scoring criteria to define the level of E-cadherin expression. Also, cut-off levels for reduced E-cadherin expression varied from 5% to 25% without evaluation standards. Finally, significant heterogeneity existed in our study. Although subgroup analyses were conducted, the results do not completely explain the observed heterogeneity.

In conclusion, this study systematically and comprehensively evaluates the prognostic value of decreased E-cadherin expression in ovarian cancer. Poor OS and PFS are significantly related to decreased expression of E-cadherin in ovarian cancer. Decreased E-cadherin expression is correlated with some clinicopathological characteristics including residual tumor size, FIGO, E-cadherin membranous staining, and surgery. Our meta-analysis demonstrates that decreased expression of E-cadherin can be a predictive biomarker of poor prognosis and a critical therapeutic target for ovarian cancer patients.

## MATERIALS AND METHODS

### Search strategy

This meta-analysis was performed in accordance with the Preferred Reporting Items for Systematic Reviews and Meta-Analysis (PRISMA) guidelines [41]. We conducted a search using PubMed, EMBASE and Cochrane Library databases for original studies that evaluated the prognostic value of decreased E-cadherin expression in ovarian cancer. The last search was conducted on March 20, 2017. The search keywords were as follows: “ovarian”, “neoplasm”, “tumor”, “cancer”, “E-cadherin”, “E-CAD”, “CDH1”, “cadherin-1”, “prognostic factor”, and “survival”. Furthermore, the reference lists of related review articles were screened. This meta-analysis was conducted based on previously published articles. Therefore, ethical approval and patient consent were not required.

### Inclusion criteria

Articles were considered eligible if they met the following criteria: (1) patients diagnosed with ovarian cancer using pathological and histological examinations; (2) the level of E-cadherin expression was detected in tumor tissues; (3) original full articles published in English; and (4) articles reported hazard ratio (HR) value and 95% confidence intervals (CIs) directly or calculated from demographic data or survival curves. Studies with more details and larger sample sizes were only selected if duplicate data from other articles occurred. Reviews, letters, conference abstracts, and comments were excluded.

### Quality assessment

The Newcastle-Ottawa quality assessment scale (NOS) was used to evaluate the quality of included studies by two independent investigators. Three perspectives (selection, comparability, and outcomes) were assessed in accordance with NOS. The quality scores of articles ranged from 0 to 9.0: scores ≥ 7.0 indicate high quality.

### Data extraction

According to the inclusion criteria listed above, two independent researchers (Lili Yu and Xiaoli Hua) extracted the following data: the first author, nation, year of publication, mean age of the patients, FIGO stage, case number, mean follow-up period, cut-off level, detecting method, survival type, method for HR estimation, HR with 95%CIs, histological type, and pathologic type. We resolved any inconsistencies through negotiation and discussion.

### Statistical analysis

Stata Version 12.0 (Stata Corporation, College Station, TX, USA) was used for statistical analysis. HR with 95%CIs was used to evaluate the prognostic value of decreased E-cadherin expression in ovarian cancer. HR estimation for OS and PFS were directly obtained in some articles. For the studies displaying survival rates with *p* values from log-rank tests or Kaplan-Meier survival curves, HR could also be extrapolated using the method of Parmar and Tierney [42]. The Chi-square-based *Q* test and *I^2^* were applied as the assessment of heterogeneity across the studies. An *I^2^* < 50% and *p* > 0.05 were considered to be homogeneity, in which case the fixed-effect model was used for analysis. If severe heterogeneity was present at *I^2^* > 50% or *p* < 0.05, the random-effect model was used. Subgroup analysis and meta-regression were conducted to explore the source of the heterogeneity across the studies [43]. Visual inspection of the funnel plot and Egger's test were done to evaluate the publication bias (*p* < 0.05 was statistically significant) [44, 45]. Sensitivity analysis was carried out to evaluate the robustness of the pooled results by sequential omission of individual studies. If univariate and multivariate analyses were both obtainable, the latter was chosen.
