# Correction: An updated meta-analysis of the prognostic value of decreased E-cadherin expression in ovarian cancer

**DOI:** 10.18632/oncotarget.25222

**Published:** 2018-04-13

**Authors:** LiLi Yu, Xiaoli Hua, Yu Yang, Ke Li, Qilin Zhang, Lixiu Yu

**Affiliations:** ^1^ Department of Obstetrics and Gynecology, Union Hospital, Tongji Medical College, Huazhong University of Science and Technology, Wuhan 430022, China; ^2^ Department of Pharmacy, Union Hospital, Tongji Medical College, Huazhong University of Science and Technology, Wuhan 430022, China

**This article has been corrected:** The correct author affiliations are given below:

^1^ Department of Obstetrics and Gynecology, Union Hospital, Tongji Medical College, Huazhong University of Science and Technology, Wuhan 430022,China

^2^ Department of Pharmacy, Union Hospital, Tongji Medical College, Huazhong University of Science and Technology, Wuhan 430022, China

Original article: Oncotarget. 2017; 8:81176-81185. https://doi.org/10.18632/oncotarget.20885

